# Evaluation of the osteoconductive potential of *Croton lechleri* sap associated with collagen or wax-based carriers in critical-size bone defects in rat calvaria

**DOI:** 10.1590/acb415926

**Published:** 2026-10-12

**Authors:** Diego Armando Leite Carvalho, Gustavo Sousa Marques, Yanna Ribeiro Macêdo, Igor Iuco Castro-Silva

**Affiliations:** 1 Universidade Federal do Ceará – Postgraduate Program in Dentistry – Fortaleza (CE), Brazil.; 2 Universidade Federal do Ceará – Dental School – Sobral (CE), Brazil.

**Keywords:** Croton, Gelatin Sponge, Absorbable, Waxes, Bone Regeneration, Biocompatible Materials, Tissue Scaffolds

## Abstract

**Purpose:**

To evaluate bone repair in critical size calvarial defects (8 mm) in rats treated with *Croton lechleri* sap (dragon’s blood sap, DBS) combined with Hemospon^®^ and Alveolex^®^.

**Methods:**

Seventy-two Wistar rats were divided into six groups: G1, Hemospon^®^; G2, Hemospon^®^ + DBS; G3, Alveolex^®^; G4, Alveolex^®^ + DBS; C−, blood clot; and C+, LuminaCoat^®^ membrane. At 30 and 60 days, specimens were submitted to histological and histomorphometric analysis, quantifying newly formed bone, residual biomaterial, and connective tissue.

**Results:**

No statistically significant differences were observed among groups in new bone formation. At 30 days, G3 presented the highest mean of newly formed bone (18.6 ± 6.8%), followed by G2 (16.0 ± 4.8%). G4 presented the lowest mean (10.7 ± 1.3%). At 60 days, C+ exhibited the greatest bone formation (29.5 ± 21.0%). G2 showed the highest biomaterial persistence at 30 (19.4 ± 0.6%) and 60 days (17.4 ± 3.9%). G3 presented the lowest connective tissue density at 30 days (49.2 ± 6.3%); C+ exhibited the lowest value at 60 days (38.9 ± 18.1%).

**Conclusion:**

Hemospon^®^ associated with DBS proved to be the most promising osteoconductive system, favoring biomaterial persistence and reducing fibrogenesis, whereas the association with Alveolex^®^ was less favorable. The DBS shows biotechnological potential, but its stabilizing effect on the scaffold requires further investigation.

## Introduction

The rehabilitation of extensive bone defects remains one of the greatest clinical challenges in oral and maxillofacial surgery, implant dentistry, and periodontics, particularly because the reconstruction of substantial losses depends on strategies capable of reconciling structural stability, tissue integration, and biological predictability^1,2^. Although autogenous bone grafting is widely recognized as the gold standard, owing to its osteogenic, osteoinductive, and osteoconductive properties, clinical limitations such as donor site morbidity and restricted volume availability drive the search for alternative biomaterials in bone tissue engineering^2-4^. In this context, the guided bone regeneration (GBR) technique has become established as a fundamental approach, particularly in the management of critical-size defects (CSD), defined as lesions whose extent exceeds the organism’s spontaneous healing capacity, resulting in predominant filling by non-mineralized fibrous connective tissue in the absence of intervention^5,6^. For the preclinical evaluation of such biomaterials and regenerative strategies, the 8-mm calvarial defect model in Wistar rats is widely employed due to its reproducibility, anatomical accessibility, and scientific validation^3,6^.

For the success of GBR, it is imperative to employ devices that combine biocompatibility, the capacity to maintain the regenerative space, stabilize the clot, and favor the orderly progression of repair^7^. Studies on resorbable membranes and carriers demonstrate that the efficacy of these systems depends directly on the interaction between their biofunctionality, residence time within the defect, and compatibility with the chronology of osteogenesis^4,8,9^. In routine dental practice, commercial devices such as Hemospon^®^ (a resorbable hemostatic sponge composed of lyophilized porcine gelatin) and Alveolex^®^ (an alveolar dressing based on propolis and iodoform) are widely used for surgical bed protection, bleeding control, and initial support of repair^10-12^. However, although clinically useful, these materials present limitations when applied in isolation in CSD, tending to act predominantly as passive physical supports or anti-infective agents, lacking the direct biological stimuli required for complete bone regeneration^3,13,14^.

In this scenario, biodiversity offers a phytochemical heritage of considerable biotechnological interest for addressing this gap with *Croton lechleri* Muell. Arg. (Euphorbiaceae) sap, popularly known as dragon’s blood sap (DBS)^15^. This natural resin is notable for a composition rich in polyphenols, condensed tannins, proanthocyanidins, and alkaloids, to which potent healing, anti-inflammatory, antioxidant, and antimicrobial activities are attributed^16-18^. Among its constituents, the alkaloid taspine is recognized as one of the main modulators of wound healing, stimulating cell migration, fibroblast chemotaxis, and the initial organization of repair tissue^19-22^. Recent investigations have focused on incorporating this phytotherapeutic agent into carrier systems and biomedical formulations, broadening the prospects for its application in local delivery platforms and modern functional biomaterials^23,24^.

Despite the sap’s considerable potential, investigations regarding its association with three-dimensional matrices in orthotopic bone regeneration models remain scarce in literature. Previously, in a heterotopic approach, Carvalho et al.^25^ incorporated DBS into alginate microspheres implanted in the subcutaneous tissue of mice, demonstrating excellent local biocompatibility, a non-irritating histological profile, and gradual dose-dependent degradation of the bioactive compound, with maintenance of systemic antioxidant potential. The preliminary validation of biological safety underpins the scientific advancement required to explore the biological behavior of the phytocomplex directly in mineralized tissue^26^.

However, the local release kinetics of this bioactive compound and the cellular response at the recipient site depend strictly on physicochemical compatibility with the vehicle employed, occurring through simple diffusion mechanisms or the biodegradation rate of the matrix^25,27^. These factors tend to vary considerably according to the structural nature of the carriers evaluated in this study: the porous, hydrophilic architecture of the gelatin sponge (Hemospon^®^) or the waxy, hydrophobic consistency of the alveolar dressing (Alveolex^®^). Therefore, investigating the incorporation of *C. lechleri* sap into these two commercial systems allows to understand how interaction with different matrices can modulate the confining microenvironment and influence the tissue repair process in CSD.

Considering the above, the present study aimed to evaluate, by means of histomorphometric analysis, bone repair in critical size calvarial defects in rats treated with *C. lechleri* sap associated with different carriers. It sought to compare the biological functionality of these associations and provide experimental grounds for the development of innovative alternatives in regenerative dentistry.

## Methods

### Ethical and legal aspects

The present study was conducted in strict compliance with international ethical principles of animal welfare, grounded in the 3Rs principles (Replacement, Reduction, and Refinement) and in accordance with the Animal Research: Reporting of In Vivo Experiments (ARRIVE) 2.0 guidelines^28^. The experimental protocol was submitted to and approved by the Animal Use Ethics Committee of the Universidade Federal do Ceará (UFC), Sobral *campus*, Ceará, Brazil, under protocol CEUA-UFC No. 03/23.

### Samples

*Croton lechleri* sap, popularly known as dragon’s blood sap (DBS), was obtained in pure commercial form (Laszlo^®^, MG, Brazil). This supplier was selected based on preliminary bioprospecting results conducted by the research group, which indicated low acute toxicity in the *Artemia salina* model (24-h LC_50_ > 22,000 µg/mL), a Raman spectral signature and phytochemical profile compatible with the presence of taspine and polyphenols^27^, in addition to regulatory approval by the Brazilian National Health Surveillance Agency (ANVISA No. 2.03740-2). DBS was then associated with two distinct carrier systems: the lyophilized porcine gelatin hemostatic sponge Hemospon^®^ (Maquira, PR, Brazil; G1), and the propolis-based alveolar dressing Alveolex^®^ (Biodinâmica, PR, Brazil; G3). Following pilot tests aimed at evaluating adherence and permeability, and a proportion of two drops (100 µL) of DBS was established as optimal for impregnating Hemospon^®^ (G2) and Alveolex^®^ (G4). The resorbable bovine type I collagen membrane Lumina Coat^®^ (Criteria, SP, Brazil; C+) was used as a collagen membrane comparator relative to the other carriers (Fig. 1).

**Figure 1:**
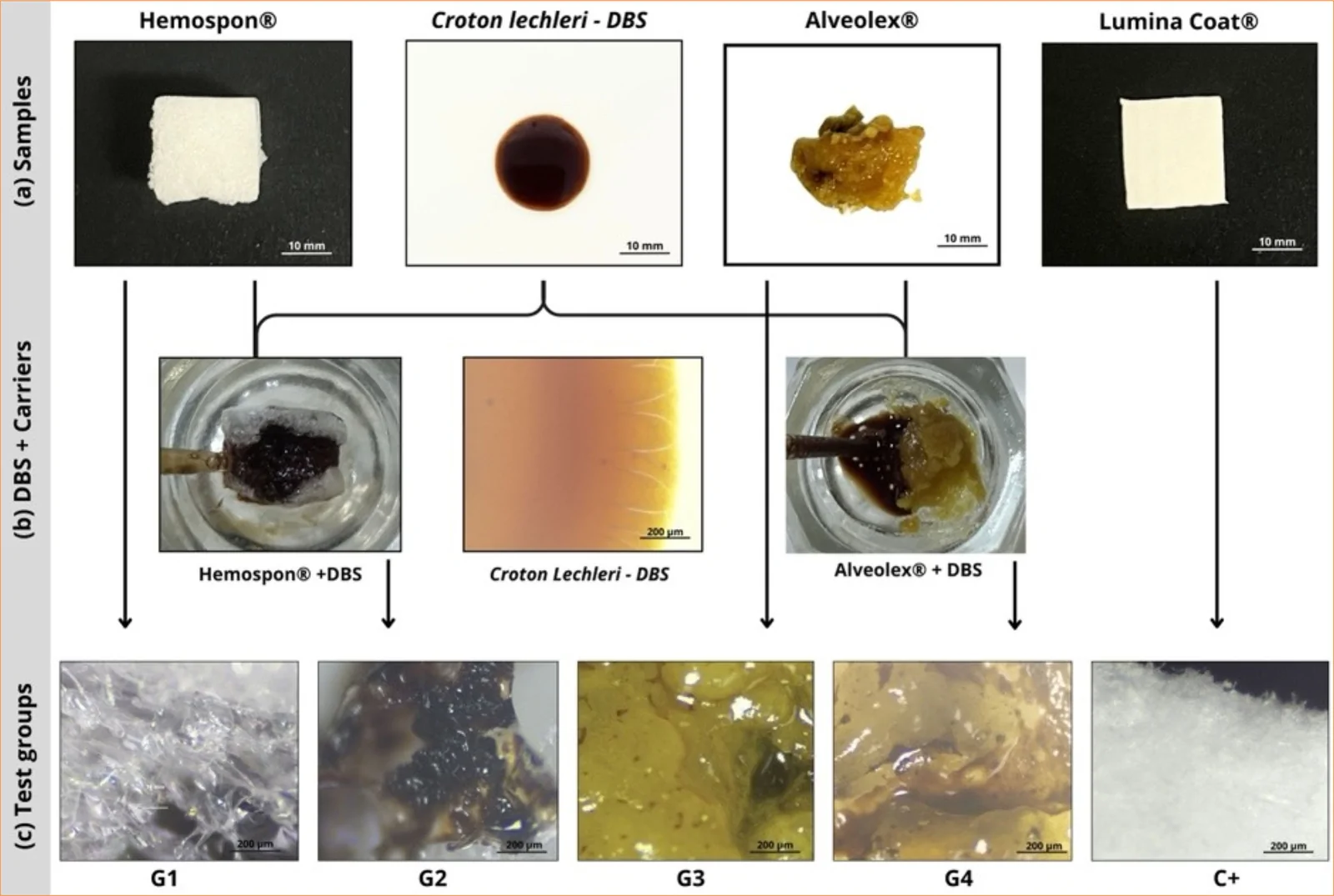
Biomaterials and positive control. (a) Macroscopic appearance; (b) Combination of dragon’s blood sap (DBS). with the carriers; (c) Microscopy of the test groups: G1 (Hemospon^®^), G2 (Hemospon^®^ + DBS), G3 (Alveolex^®^), G4 (Alveolex^®^ + DBS), and C+ (Lumina Coat^®^).

Source: Elaborated by the authors.

### Critical-size defect implantation in Wistar rats

The experimental model employed male Wistar rats (*Rattus norvegicus albinus*) weighing between 200 and 220 g. The animals were housed in collective cages according to experimental group at the animal facility of UFC’s Sobral *campus*, under controlled temperature and a 12-hour light/dark cycle, with water and standard chow available *ad libitum*. Seventy-two animals were distributed according to the different experimental conditions, considering six groups at 2 time points, with six animals per group.

The CSD model was employed, characterized by the absence of spontaneous healing throughout the animal’s lifetime. The surgical protocol began with intraperitoneal anesthesia, administered using 10% ketamine solution (Dopalen^®^, Sespo Indústria e Comércio LTDA, SP, Brazil) at a dose of 80 mg/kg, and 2% xylazine (Anasedan^®^, Sespo Indústria e Comércio LTDA, SP, Brazil) at a dose of 10 mg/kg. This was followed by trichotomy of the upper portion of the head and antisepsis with aqueous 2% chlorhexidine (Rioquímica LTDA, SP, Brazil). A semilunar incision was then made in the skin of the frontoparietal region of the skull using a No. 15 scalpel blade. After elevation of the mucoperiosteal flap with a Molt periosteal elevator, the bone cortex was exposed. Subsequently, a circular craniectomy of 8 mm in diameter was performed to create the CSD, using a trephine bur (Sistema de Implante Nacional, Brazil) at a 20:1 reduction ratio (Dentscler, Brazil) mounted on a surgical micromotor (VK Driller Equipamentos Elétricos LTDA, Brazil), under abundant irrigation with cold, sterile saline solution (0.9% NaCl) throughout the procedure.

The osteotomized bone disc was carefully removed with the aid of a No. 1 Ochsenbein chisel. Finally, each defect was filled according to the sham group, consisting of the defect filled only with a blood clot (C−), or with one of the five therapeutic modalities established in the experimental design (Fig. 2).

**Figure 2:**
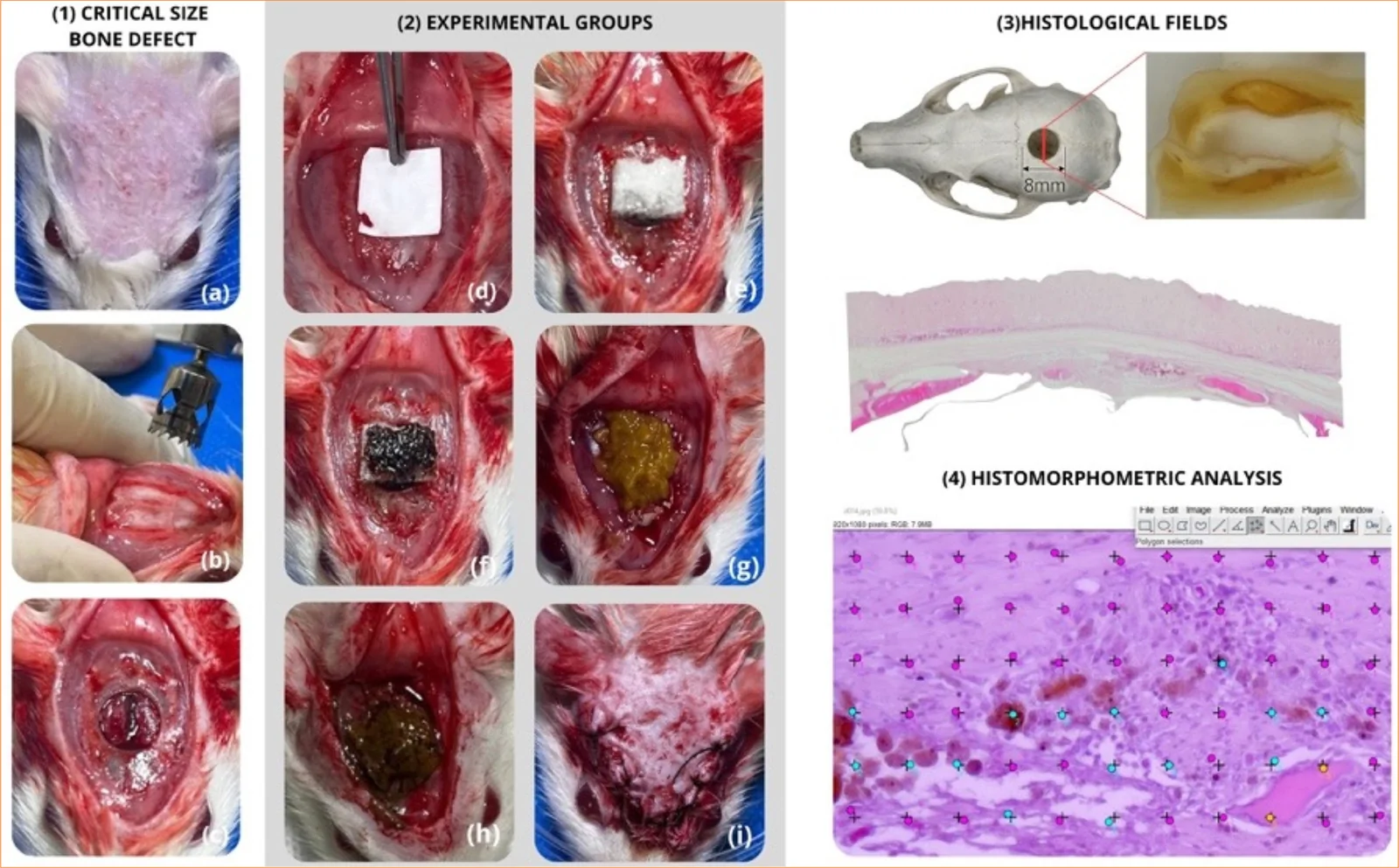
*In-vivo* evaluation of osteoconduction. (1) Creation of the critical-size defect: (a) hair removal; (b) osteotomy using an 8-mm trephine; (c) bone defect (C−). (2) Implantation in experimental groups: (d) C+ (Lumina Coat^®^); (e) G1 (Hemospon^®^); (f) G2 (Hemospon^®^ + DBS); (g) G3 (Alveolex^®^); (h) G4 (Alveolex^®^ + DBS); (i) suturing. (3) Coronal sectioning and processing for histological field capture. (4) Histomorphometric analysis via point counting using ImageJ software.

Source: Elaborated by the authors.

The mucoperiosteal flap was repositioned and sutured in layers using 4-0 nylon suture (Procare^®^, Medico Industries & Trade Co-Shijiazhuang, China). During the immediate postoperative period, a preemptive and maintenance analgesia protocol was instituted, consisting of subcutaneous administration of an anti-inflammatory agent every 12 hours for two days. The animals were systematically monitored for clinical and systemic parameters to ensure their well-being.

Euthanasia was performed at the experimental time points of one and two months after the intervention, by intraperitoneal overdose of anesthetic solution. Immediately after collection, specimens were fixed in 10% buffered formalin (v/v, pH 7.0) for 48 hours. Following fixation, all necropsy specimens were decalcified in an acid decalcifying solution (Allkimia, Brazil) for 72 h, rinsed in running water for 1 h, sectioned with a blade through the center of the bone defect in the longitudinal plane, and submitted to conventional histological processing: dehydration in an increasing ethanol series (70% to 100%), clearing in xylene, and paraffin embedding.

Serial 4-µm sections were obtained using a microtome and stained with hematoxylin and eosin (H&E). Qualitative and descriptive histopathological evaluation was conducted under the supervision of an experienced pathologist.

### Histomorphological and histomorphometric analysis

The qualitative analysis consisted of evaluating the bone repair pattern, connective tissue organization, and persistence of the grafting biomaterials. For documentary purposes, representative photomicrographs covering the entire extent of the defect were captured using a DI-260 biological microscope (Digilab, Piracicaba, SP, Brazil) coupled to a high-resolution digital camera (48 megapixels, model NewValue, Panasonic, São Paulo, SP, Brazil).

For the quantitative analysis, contiguous, non-overlapping fields—ranging from 5 to 7 fields per histological section—were evaluated to encompass the entire critical defect. Image processing was performed using ImageJ software, version 1.8.0 (National Institutes of Health, United States of America), duly calibrated to the dimensions of the captured images. The biological parameters mentioned above were counted using a 112-point grid overlaid on each photomicrograph, and based on the absolute number of points obtained, the percentage volume density (%i) of each parameter was determined, according to Eq. 1:


(1) %i=(pi)100%/P

where: *pi*: the number of points corresponding to the parameter; P: the total number of points.

Raw data were tabulated in Excel (Microsoft Office, United States of America), graphically expressed as mean ± standard deviation, and statistically analyzed using Jamovi software, version 2.3.28 (Jamovi Project, Australia), for comparisons between groups according to the experimental criteria and time points.

### Statistical analysis

Data were tabulated and expressed as mean of standard deviation. Normality of distribution was verified, and for intergroup comparisons within the same time, one-way analysis of variance (ANOVA) was applied, followed by Tukey’s post hoc test. Differences were considered significant at *p* < 0.05 (95% confidence level).

## Results

Histological and histomorphometric analyses were performed in critical-size bone defects in rat calvaria, using the following experimental groups:

- G1: Hemospon^®^;
- G2: Hemospon^®^ associated with DBS;
- G3: Alveolex^®^;
- G4: Alveolex^®^ associated with DBS;
- C−: blood clot as negative control;
- C+: LuminaCoat^®^ membrane as positive control.

Histological analysis revealed, in all groups at the first month, newly formed bone predominantly at the defect margins, adjacent to the native cortical bone, with less pronounced bone islands in the central region, a pattern consistent with centripetal osteogenic growth. In the second month, progression of osteogenic activity was observed, with expansion of the bone islands, more evident in C+, G1, and G3. Connective tissue was more abundant in C−, evolving from a loose extracellular matrix in one month to a more fibrous tissue in the second month, whereas C+ exhibited a significant reduction. Residual biomaterial was observed in all treated groups at both experimental time points, with G2 showing greater persistence and G1 exhibiting more pronounced degradation between the evaluated intervals (Fig. 3).

**Figure 3:**
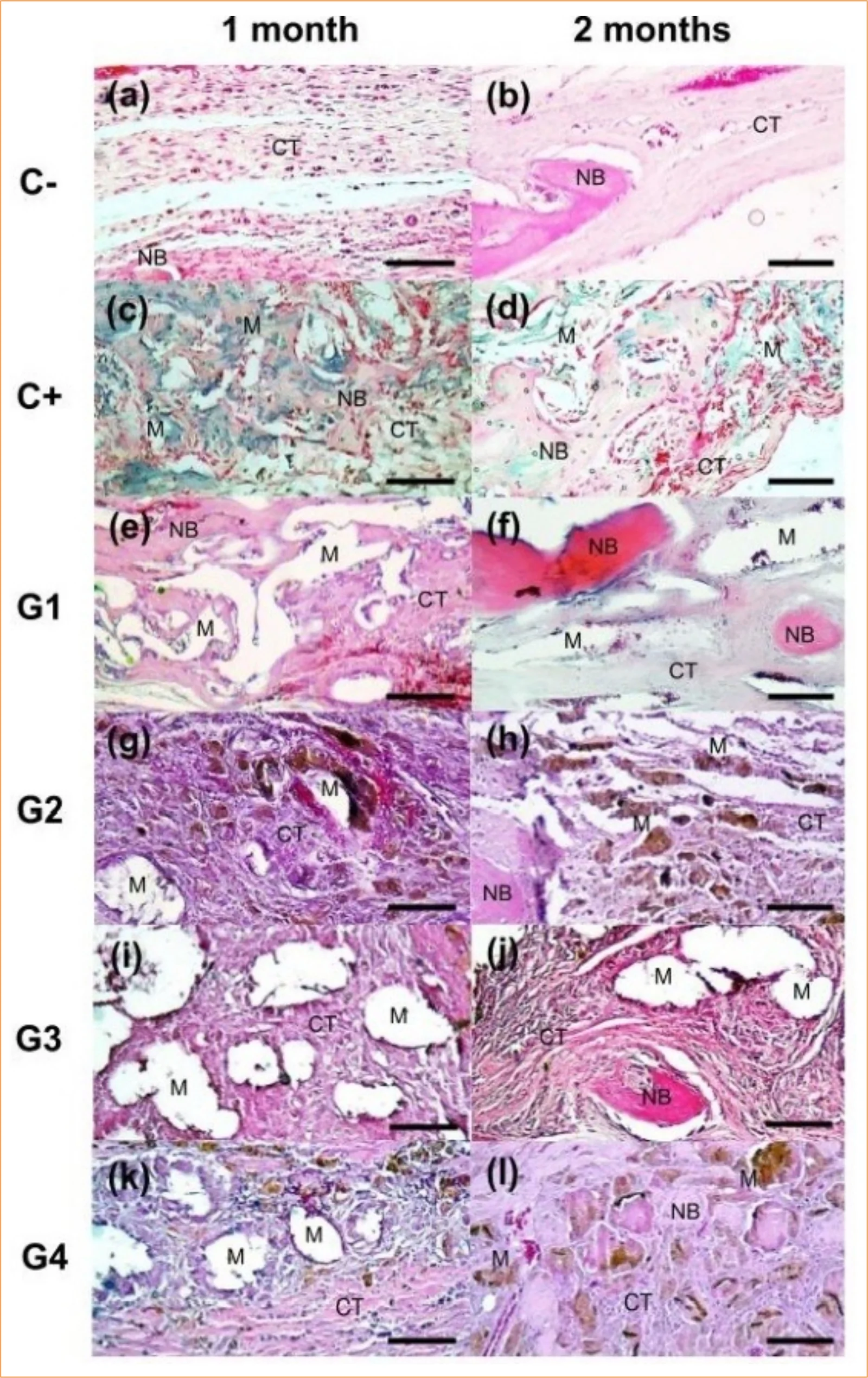
Histological analysis of osteoconduction in a critical-sized bone defect in rat calvaria at one and two months. Groups: (a and b) C− (blood clot), (c and d) C+ (Lumina Coat^®^), (e and f) G1 (Hemospon^®^), (g and h) G2 (Hemospon^®^ + DBS), (i and j) G3 (Alveolex^®^), and (k and l) G4 (Alveolex^®^ + DBS). Staining: hematoxylin-eosin. Magnification: 100×. Scale bar: 100 µm.

CT: connective tissue; NB: newly formed bone; M: residual material. Source: Elaborated by the authors.

Some findings in the microscopic analysis revealed intriguing aspects. Among these, C+ showed intense basophilia and metachromasia within the biomaterial matrix, indicating acidification and active enzymatic degradation. At 30 days, a marked resorption kinetics of the implanted material was observed, which was attenuated at 60 days by a morphological transition in which the residual biomaterial became more integrated into the newly formed matrix. Another interesting aspect was observed in G1, in which the hemostatic effect of the Hemospon^®^ sponge could be seen, followed by erythrocyte agglutination, a phenomenon that favors the healing process.

At the first month, the volumetric density of newly formed bone showed no statistically significant differences among groups (*p* > 0.05), although substantial numerical variations were observed. G3 stood out with the highest mean for the period (18.57 ± 6.75%), followed by G2 (16.03 ± 4.75%), C+ (14.15 ± 6.15%), G1 (12.57 ± 6.63%), C− (12.28 ± 6.97%), and G4 (10.70 ± 1.29%), which recorded the lowest initial mean among all groups. At two months, the bone neoformation profile underwent reorganization among the groups. C+ then presented the highest mean for the period (29.45 ± 21.02%), representing a substantial increase relative to the previous month, although it did not reach statistical significance by Tukey’s criterion (*p* > 0.05). In the intergroup analysis, comparisons revealed no significant differences between C+ and G1 (17.71 ± 4.19%), G2 (14.22 ± 2.04%), G3 (18.65 ± 11.23%), C− (14.00 ± 5.33%) or G4 (11.29 ± 3.35%). Among the carriers, G2 and G3 sustained the highest means at 60 days, whereas G4 maintained the lowest mean at both time points, with no group showing a statistically significant difference between the experimental time points (Fig. 4a).

**Figure 4:**
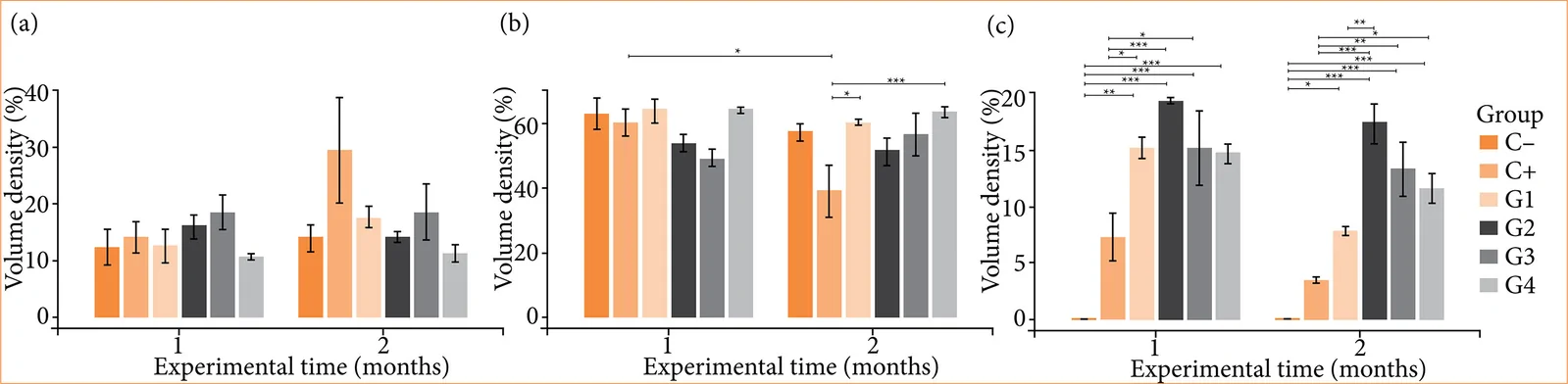
Histomorphometric analysis of the volumetric density of (a) new bone, (b) connective tissue, and (c) biomaterial.

*p=0.01–0.04; **p=0.001–0.009; ***p<0.001. Source: Elaborated by the authors.

In the connective tissue analysis at the first month, volumetric density showed no statistically significant differences among the experimental and control groups (*p* > 0.05), with decreasing scores for G4 (64.04 ± 1.85%), G1 (63.94 ± 7.98%), C− (62.70 ± 11.33%), C+ (60.29% ± 9.09%), G2 (54.02 ± 6.00%), and G3 (49.21 ± 6.31%). In two months, C+ (38.89 ± 18.09%) showed a significant reduction in density equivalent to 35% of its initial volume, establishing itself as the lowest mean of study, with statistically significant differences compared with G4 and G1. In contrast, C– (57.54 ± 5.30%), G1 (60.55 ± 1.39%), G3 (56.54 ± 14.70%), and G4 (63.51 ± 4.18%) maintained the highest connective matrix densities at the end of the experiment, suggesting that both interventions favored the persistence of this tissue in spaces not occupied by bone. G2 (51.02 ± 9.92%) exhibited lower density compared to the other experimental groups, suggesting that association with DBS may have modulated the healing response relative to Hemospon^®^ alone, keeping fibrous tissue volume at lower levels than those observed in G1 (Fig. 4b).

At both experimental time points, C− showed no measurable biomaterial, an expected finding given the absence of implanted material. At the first month, the volumetric density of residual biomaterial did not differ significantly among the treated groups (*p* > 0.05 for all intergroup comparisons), with the greatest persistence observed in G2 (19.44 ± 0.59%), followed by G3 (15.27 ± 7.54%), G1 (15.18 ± 2.10%), and G4 (14.74 ± 1.84%), whereas C+ (7.25 ± 4.86%) exhibited the lowest mean, showing a significant difference relative to G1 (*p* = 0.030), G2 (*p* < 0.001), and G3 (*p* = 0.027).

At two months, despite the reduced presence of residual biomaterial observed in all treated groups, especially in C+ (3.43 ± 0.58%) and G1 (7.77 ± 0.91%), with reduction in density equivalent to 53 and 48% of its initial volume, respectively, there were no significant differences between time points for any individual group (*p* > 0.05). In the intergroup analysis at the second month, G2 (17.44 ± 3.93%) stood out for the greatest biomaterial persistence, being higher than C+, G1, and G4, whereas G3 (13.38 ± 5.43%) and G4 (11.63 ± 2.94%) maintained stable profiles with no significant differences between each other or relative to the other groups (Fig. 4c).

## Discussion

Bone tissue formation in CSD constitutes a multifactorial biological process that requires the systematic sequence of cellular and molecular events, including chemotaxis, proliferation, differentiation of osteoprogenitor cells, and maturation of the calcified extracellular matrix^22^. In the present study, we investigated whether *C. lechleri* sap (DBS), when associated with distinct biotechnological carriers, such as the lyophilized porcine gelatin sponge Hemospon^®^ (G2) and the propolis-based alveolar dressing Alveolex^®^ (G4), would be capable of potentiating osteoconduction in a critical-size bone defect in the calvaria of Wistar rats, thereby transforming these carriers from passive barriers into bioactive delivery systems with a modulatory action on GBR.

The 8-mm diameter CSD model in the calvaria of Wistar rats was adopted as the experimental protocol due to its recognized reproducibility and broad consolidation in the literature for the analysis of regenerative protocols^3,6,29^. The CSD corresponds to a defect that does not heal spontaneously throughout the animal’s lifetime. The choice of this model precludes any bias arising from spontaneous healing, ensuring that any intergroup differences are attributable exclusively to the test biomaterials. Likewise, the decision to adopt one- and two-month intervals in this study was based on their representing distinct and complementary biological windows, allowing the capture of events ranging from early osteogenesis to the maturity and consolidation of mineralized tissue, in accordance with the evaluation timeline established in previous studies^3,8,9,14,30^.

Group C− filled exclusively with a blood clot showed no statistically significant increase in the volumetric density of newly formed bone between the first and second months of evaluation. The values found fell within the widely documented range of variability for critical defects without biomaterial intervention, which ranges from 4% in the first month^2^, to 9.81% in the second month^7^, and up to 20% in the third month^31^. The stability of bone neoformation in C− reinforces the concept that calvaria’s intrinsic osteogenic potential is insufficient to promote defect closure, keeping it predominantly filled with loose connective tissue at the first month, which evolves into a more organized fibrous profile by the second month^7,29,32^.

Group C+ treated with the bovine collagen membrane (Lumina Coat^®^) exhibited an ascending trajectory of bone neoformation between the first and second months, accompanied by marked intragroup variability at the final time point. This statistical dispersion and the late incremental repair kinetics are attributed to the heterogeneous distribution of its fibers and the variable porosity of the natural matrix, characteristics that compromise barrier stability and limit control over its in-vivo resorption rate^8,30,33^, a pattern of methodological heterogeneity corroborated in the literature for calvarial models^34,35^. In this study, C+ exhibited the lowest degradation kinetics among all groups, with statistically significant differences relative to G1, G2, and G3. Simultaneously, this group presented the greatest reduction in connective tissue density, reinforcing the effectiveness of the biomaterial in the biological principle of cell exclusion and mechanical occlusion of non-osteogenic lineages within an ideal porosity window^30,31,36^.

Although the Lumina Coat^®^ collagen membrane presents osteoconductive properties^31^, its biological functionality may be optimized by association with DBS, transitioning this scaffold into a bioactive system capable of modulating the recruitment and differentiation of osteoprogenitor cell lineages^37^. This approach is grounded in the contemporary paradigm of third-generation biomaterials, which advocates spatiotemporal control over the release of bioactive factors from functionalized scaffolds, a process that supports the molecular signaling required to orchestrate cellular responses throughout the sequential phases of osteogenesis^4,37^. In this context, DBS, which already presents a complex phytochemical profile encompassing the alkaloid taspine, proanthocyanidins, polyphenols, and diterpenes, stands out as a promising bioactive candidate for the functionalization of three-dimensional scaffolds aimed at the repair of critical-size bone defects^15^.

G1 showed a progressive increase in bone neoformation between the first and second months of evaluation, indicating that the material provided an initial contribution to defect stabilization and repair organization. This behavior is consistent with the general properties attributed to porous hemostatic sponges, which favor fluid absorption, clot retention, and temporary physical support to the surgical bed^12^. However, the persistence of high connective tissue densities throughout the experiment, associated with marked degradation of the residual biomaterial by the second month, suggests that this initial support was not sufficient to sustain a more effective reparative progression in CSD. This finding is consistent with the GBR literature, according to which the efficacy of resorbable membranes and carriers depends not only on their biocompatibility but also on their capacity to maintain space and present biofunctionality compatible with the chronology of osteogenesis^2,4,8^.

Studies in critical calvarial defects have also demonstrated that passive or rapidly resorbing biomaterials tend to show limited performance when not associated with additional osteopromotive properties, favoring the persistence of connective tissue in spaces not converted into bone^3,9,14^. In this sense, although Hemospon^®^ showed utility as an initial hemostatic carrier, the behavior observed in G1 reinforces that its isolated action appears insufficient to promote more robust bone repair in CSD, which justifies investigating its association with bioactive agents capable of modulating material degradation and biologically enriching the regenerative microenvironment.

G2, treated with Hemospon^®^ associated with DBS, exhibited a distinct reparative profile, particularly with greater biomaterial persistence throughout the experimental period, accompanied by a downward trend in connective tissue between the first and second months of evaluation. This decreasing trend in connective matrix density in G2 suggests that DBS favorably modulated the local healing response compared with Hemospon^®^ alone. This behavior corroborates previous findings from heterotopic subcutaneous biocompatibility testing in mice, in which incorporation of the sap into a hydrophilic alginate matrix mimicked this profile of low tissue irritation and controlled resorption^25^. Thus, the slow release of bioactive compounds from hydrophilic, porous matrices appears to attenuate the chronic inflammatory response and limit local fibrogenesis, optimizing the microenvironment for guiding repair.

The hypothesis regarding G2 is supported by the chemical composition of *C. lechleri*, rich in polyphenols, tannins, proanthocyanidins, and taspine, compounds related to healing, antioxidant, and tissue repair-modulating activity^15–18^. Taspine, in turn, has been described as an important mediator of wound healing, stimulating cell migration and the organization of repair tissue^19,20^. In addition, experimental studies have shown that these bioactive compounds may favor osteoblastic activity and mineralization, broadening the biological plausibility of their application in bone repair^38^. Since porous hemostatic sponges may act as local reservoirs of fluids and bioactive compounds, contributing to clot stability and to the maintenance of the regenerative space, it is possible that the association between DBS and Hemospon^®^ favored a more suitable microenvironment for regeneration^4,12^.

Although this interaction was not directly measured in the present experimental design, a previous biocompatibility study in murine subcutaneous tissue, using alginate microspheres associated with the same DBS, demonstrated partial dose-dependent biodegradation and a non-irritating histological pattern, with no induction of an exacerbated fibrotic response^25^. This finding, obtained under experimental conditions directly comparable in terms of sap origin, reinforces the plausibility that DBS modulates the degradation kinetics of porous, hydrophilic matrices, a pattern also described for other carrier systems incorporating *C. lechleri* ^23,24^. Thus, the greater persistence of the material in G2 may represent a biotechnological advantage by better aligning carrier biodegradation with the chronology of bone neoformation in critical defects.

These data indicate that incorporating DBS into Hemospon^®^ significantly altered the degradation kinetics of the scaffold. In this sense, DBS emerges as a candidate stabilizing agent for grafts with rapid degradation. As demonstrated by Sousa et al.^39^, bone allografts and anorganic bovine xenografts used in GBR exhibit distinct bioresorption and osteoconduction kinetics, with variations that may compromise clinical outcomes when degradation precedes osteoblastic repopulation. In this scenario, the incorporation of bioactive compounds such as DBS into such carriers, aimed at modulating their degradation rate and enriching the regenerative microenvironment with chemotactic signals for osteoprogenitor cells, constitutes a promising perspective that warrants systematic investigation in models integrating microtomographic analysis and dynamic histomorphometry.

G3, treated with Alveolex^®^ alone, exhibited a consistent and stable regenerative profile throughout the experimental period. At the first month, it recorded the highest mean volumetric density of newly formed bone, maintaining the same value at two months, suggesting early stabilization of the mineralization kinetics. In parallel, this group presented relatively stable connective tissue densities between the evaluated periods, accompanied by a slow, discreet degradation of the residual biomaterial. These results align with the findings of Pereira et al.^10^, who observed 11.45% bone neoformation at 30 days in defects treated with Alveolex^®^, albeit in a distinct experimental model.

Thus, the numerically favorable behavior of G3 suggests a possible bioactive and osteoconductive contribution of the alveolar dressing to repair in CSD. The literature attributes to propolis, the active component of Alveolex^®^, anti-inflammatory, antioxidant, and osteomodulatory properties related to the presence of compounds such as caffeic acid phenethyl ester, quercetin, and phenolic acids, which modulate pathways associated with attenuation of osteoclastogenesis and stimulation of osteoblastic differentiation^40,41^. Although these molecular mechanisms were not directly evaluated in the present study, they provide a biological rationale for the histomorphometric performance observed in G3. Furthermore, the well-established clinical use of Alveolex^®^ in the prevention of dry socket is based on its local hemostatic and anti-inflammatory effect, properties that possibly contributed to optimizing the initial repair microenvironment^42^.

Contrary to the initial hypothesis of phytochemical synergy, G4 (Alveolex^®^ + DBS) showed the least favorable behavior regarding bone neoformation, with the lowest means observed at both experimental time points. A plausible hypothesis for this finding is the existence of physicochemical incompatibility between DBS and the waxy base of Alveolex^®^. DBS predominantly presents polar nature, with a composition rich in polyphenols and other water-soluble bioactive compounds^15,16,18^, whereas Alveolex^®^ has a waxy, hydrophobic composition. This difference may have hindered homogeneous incorporation of the sap into the dressing, compromising the local distribution of bioactive compounds and, consequently, the biological performance of the association. In contrast, the relatively more favorable behavior of G3, treated with Alveolex^®^ alone, is consistent with the literature attributing to propolis anti-inflammatory, antioxidant, and osteomodulatory properties, including effects on osteoblastic activity and modulation of bone resorption^10,38,41^. This finding suggests that the inferior performance of G4 did not stem from a lack of bioactivity of Alveolex^®^ itself, but possibly from an unfavorable interaction between its components and DBS. In turn, the literature indicates that *C. lechleri* sap contains compounds with recognized healing and antioxidant activity, such as taspine and proanthocyanidins, capable of modulating the early stages of tissue repair^17,20,21,27^. Thus, the divergence between the biological potential of DBS and the performance observed in G4 reinforces the importance of the carrier for long-term bioactive release. The efficacy of the sap depends not only on its intrinsic bioactivity but also on its compatibility with the carrier vehicle employed as a critical determinant of the regenerative response.

Although preclinical trials have validated the biological safety of taspine, with no carcinogenesis-promoting activity reported in long-term studies, it should be noted that the tissue repair profile in sites beyond the cutaneous bed remains poorly explored^19^. In the present experimental design, local application of a 100-µL dose of pure, concentrated sap may have saturated the recipient bed, exceeding the optimal bioactivity window favorable to osteogenesis. This phenomenon is grounded in a hormetic behavior, whereby reduced doses of *C. lechleri* sap exhibit greater *in-vitro* viability in human osteoblasts^43^, whereas higher doses exert pro-oxidant effects inhibit lymphocyte proliferation and reduce the relative phagocytosis of human leukocytes^15,44^. Therefore, the substantial persistence of fibrous connective tissue and the lower rate of bone neoformation observed suggest that the administered dose induced a paradoxically inhibitory effect on the differentiation and chemotaxis of native osteoprogenitor cells, warranting the need for standardization based on orthotopic dose-response curves.

The absence of a dose-response design encompassing different DBS concentrations associated with each carrier constitutes a methodological limitation that precludes direct confirmation of this hypothesis. This scenario reinforces the urgent need for dosage standardization, an urgency advocated by Tezza^45^, who emphasizes the use of allometric scaling to ensure that the therapeutic threshold does not compromise biological safety, based on a no-observed-adverse-effect level of 400 mg/kg in mice, 200 mg/kg in rats, and only 32.43 mg/kg for the human equivalent dose.

## Conclusion

The association of *C. lechleri* sap with the gelatin sponge configured itself as the system with the most favorable osteoconduction, demonstrating a stabilizing effect on biodegradation kinetics and a trend toward reduced local fibrogenesis. Conversely, its incorporation into the waxy-based dressing proved unfavorable to the regenerative microenvironment, resulting in the lowest osteogenic index in the study, attributed to a possible physicochemical incompatibility between the components. Although no statistically significant differences in bone neoformation were observed among the groups, the regenerative performance of the sap proved strictly dependent on compatibility with the carrier employed. Future dose-response studies are needed to elucidate the underlying mechanisms and optimize the biotechnological application of the sap in bone tissue engineering.

## Data Availability

All datasets were generated or analyzed in the present study.
